# Authors’ reply

**Published:** 2010

**Authors:** Pranab Das, Jagat Ram, G S Brar, M R Dogra

**Affiliations:** Advanced Eye Center, PGIMER, Chandigarh, India

Dear Editor,

We thank Sharma[1] for the encouraging comments on our article,[2] “Results of intraocular lens implantation with capsular tension ring in subluxated crystalline or cataractous lenses in children”. We agree with the author that in patients with zonular dialysis > 180 degrees, the Cionni-modified capsular tension ring (CTR), with a single or double eyelet inserted and sutured to the sclera, will show better results in terms of centration and long-term stability.[3] We are now inserting the Cionni ring with single or double eyelet in patients with significant subluxation, 120 degrees or more, and performing trans-scleral fixation, for better centration.
